# Hyperspectral imaging for tumor segmentation on pigmented skin lesions

**DOI:** 10.1117/1.JBO.27.10.106007

**Published:** 2022-10-31

**Authors:** Eleni Aloupogianni, Takaya Ichimura, Mei Hamada, Masahiro Ishikawa, Takuo Murakami, Atsushi Sasaki, Koichiro Nakamura, Naoki Kobayashi, Takashi Obi

**Affiliations:** a Tokyo Institute of Technology, Department of Information and Communications Engineering, Meguro, Japan; b Saitama Medical University Moroyama Campus, Department of Pathology, Faculty of Medicine, Iruma, Japan; c Saitama Medical University Hidaka Campus, Faculty of Health and Medical Care, Hidaka, Japan; d Saitama Medical University Moroyama Campus, Department of Dermatology, Faculty of Medicine, Iruma, Japan; e Tokyo Institute of Technology, Institute of Innovative Research, Yokohama, Japan

**Keywords:** gross pathology, hyperspectral, segmentation, skin lesions, medical image processing

## Abstract

**Significance:**

Malignant skin tumors, which include melanoma and nonmelanoma skin cancers, are the most prevalent type of malignant tumor. Gross pathology of pigmented skin lesions (PSL) remains manual, time-consuming, and heavily dependent on the expertise of the medical personnel. Hyperspectral imaging (HSI) can assist in the detection of tumors and evaluate the status of tumor margins by their spectral signatures.

**Aim:**

Tumor segmentation of medical HSI data is a research field. The goal of this study is to propose a framework for HSI-based tumor segmentation of PSL.

**Approach:**

An HSI dataset of 28 PSL was prepared. Two frameworks for data preprocessing and tumor segmentation were proposed. Models based on machine learning and deep learning were used at the core of each framework.

**Results:**

Cross-validation performance showed that pixel-wise processing achieves higher segmentation performance, in terms of the Jaccard coefficient. Simultaneous use of spatio-spectral features produced more comprehensive tumor masks. A three-dimensional Xception-based network achieved performance similar to state-of-the-art networks while allowing for more detailed detection of the tumor border.

**Conclusions:**

Good performance was achieved for melanocytic lesions, but margins were difficult to detect in some cases of basal cell carcinoma. The frameworks proposed in this study could be further improved for robustness against different pathologies and detailed delineation of tissue margins to facilitate computer-assisted diagnosis during gross pathology.

## 1 Introduction

In Japan, incidence and mortality rates of skin cancer have increased in the past decades, despite adjustments for an aging population.^1^ At the same time, the American Cancer Society estimates that malignant skin tumors comprise the majority of all cancers in the United States.^2^ Early diagnosis is key for the effective treatment of skin tumors. Dermoscopy and histology remain the golden standards for clinical detection and diagnosis, respectively. However, the intermediate stage of gross pathology hinders the digital flow. Gross pathology is a visual assessment stage that includes the manual preparation of *ex-vivo* tissue for histology. Despite advances in medical technology, gross pathology lacks digitization, automation, and standardization. At the same time, it is time-consuming and dependent on the skill of the pathologist. However, pigmented skin lesions (PSL), a group of pathologies with a high incidence rate, are rich in color information. Hyperspectral imaging (HSI) is a noninvasive imaging technique that can capture spectral signatures of a tissue sample, capable of discriminating malignancies.^3^ A noninvasive, nonionizing optical biopsy based on HSI could speed up diagnosis, reduce the number of re-excisions, enable telepathology, and increase treatment effectiveness.

Hyperspectral Imaging (HSI) is an imaging technique developed for remote sensing applications, which is currently an emerging modality in medical imaging.^4^^,^^5^ Through the use of narrow-band filters, HSI can capture the reflectance spectrum of a target tissue in detail, in the form of spectral signatures. These signatures function as a fingerprint of the substances that comprise the tissue, due to the relationship between reflectance and absorbance. Total absorbance is the sum of the absorbance of individual skin chromophores, guided by the Beer–Lambert law. Therefore, HSI can capture color information with more detail compared to standard RGB cameras, which suffer from metamerism phenomena. The shape of spectral signatures can be related to concentrations of chromophores. The main chromophores of the skin, melanin (M), hemoglobin (${\mathrm{HbO}}_{2}$), and deoxyhemoblobin (Hb) show increased concentrations in tumor growths, as a result of vascularization and deep melanin structures. Consequently, spectral signatures of tissue areas with tumor growths are expected to differ from healthy tissue.

The end goal of histopathology is to determine the existence or absence of malignancy and to evaluate the status of tissue margins. Knowledge of the tumor margins at an earlier stage, either during clinical examination, during surgery, or during gross pathology, can greatly enhance treatment outcomes. Optical margin detection can be interpreted as a segmentation problem of recovering healthy and tumor segments on the tissue surface. Segmentation is a recent research trend for HSI data in the medical field with limited applications as of yet.^6^ Pardo et al.^7^ segmented melanoma and nevus areas on lesions using kernel density estimation with maximum *a posteriori* estimation with good sensitivity. However, these segments were used for classification purposes and thus were not evaluated for margin detection. Leon et al.^8^ proposed K-means clustering and spectral angle mapper (SAM) similarity for the segmentation of *in-situ* PSL. However, the validation and testing dataset contained mostly melanocytic lesions, and performance was not validated against histology margins. Deep learning-based segmentation has been applied for HSI of the retina,^9^ liver,^10^ head, and neck^11^ or tongue gross tissue^12^ using a Dense fully-convolutional neural network with U-Net^13^ backbone. More work has been done in histology HSI data, either with convolutional U-Net,^14^ patch-based multiple instance learning (MIL)^15^ or linear discriminant analysis (LDA).^16^ Regarding nonmedical applications, networks that use Xception block^17^ or a three-dimensional convolutional neural network (3D CNN)^18^^,^^19^ show good performance in the segmentation of remote sensing HSI data.

The structure of HSI data cubes complicates the application of deep learning models for segmentation. Common models in artificial intelligence are prepared and optimized for RGB natural images, not HSI or medical images. In many cases, dimension reduction of an HSI to three channels is applied, before feeding the dataset to a pretrained network.^18^^,^^20^^,^^21^ However, such a process can discard notable spectral features. On the other hand, if a network that preserves the HSI’s spatio-spectral structure is designed and trained from scratch, a large number of hyperparameters need to be learned, resulting in an illposed problem for limited data samples. Alternatively, segmentation of HSI is commonly trivialized in the classification of individual spectral signatures, discarding the spatial structure. This process ignores spatial correlations in tissue and contradicts the purpose of acquiring two-dimensional (2D) HSI data in the first place. Additionally, evaluation is commonly performed in terms of specificity and/or sensitivity, which is not appropriate for segmentation tasks.^22^ A high number of correctly classified pixels of healthy tissue along with a small number of tumor pixels can skew evaluation metrics. Despite the variety of proposed methodologies for HSI data, spectral signatures of gross tissue have inherent properties that differ from HSI in remote sensing, food technology, or other data spaces. Therefore, the appropriateness of common HSI methodologies needs to be adapted for skin applications and verified accordingly.

Most studies focus on skin pathology use only melanoma versus nevus datasets, even though PSL includes a variety of pathologies. Previous studies used histology slides with longitudinal sections as ground truth labels. However, this is not a viable option for large-scale data collection and periodic upgrades in the segmentation model. Histology slides are prepared from cross-sections in standard clinical practice. Such an approach can achieve excellent training under experimental conditions but is not feasible for clinical applications. Specifically for HSI data, the large vector size (several hundreds of points for each spectral signature) complicates and hinders common models. Moreover, hospitals and clinics are usually not updated with high-performing computer systems. Therefore, a framework with balance performance and memory requirements are necessary.

These works investigate the problem of tumor margin detection on PSL during gross pathology using HSI data. The goal is to propose a framework for margin detection, which has good enough performance to facilitate computer-assisted diagnosis. This is the first work according to our knowledge to investigate this problem for *ex-vivo* gross PSL tissue samples. Performance was evaluated with emphasis on the two-dimensional structure of the predicted tumor segments and investigation of the parameters that affect model decision.

## 2 Materials and Methods

### 2.1 HSI Acquisition System

A custom acquisition system was designed, with a 2D spectroradiometer at its core. The system was developed and calibrated specifically for applications in skin gross pathology.^23^ An HSI camera (SR-5000, TOPCON Corporation, Japan; 1.4 Mpixel CCD image sensor, lens 32 mm, 1376×1024 pixel, 380 to 780 nm) was positioned above the capture base, and an LED source (SERIC Solax-iO LE-9ND55, SERIC, Japan) was arranged at 45 deg from the capture base. Two polarizers (TS High Contrast Plastic Polarizing Plate, Edmund Optics, Japan) were positioned in front of the lens and the light source, in a Crossed Nicols configuration. The system was enclosed in a dark box. The HSI data cube had spectral range (420, 750) nm with step 1 nm, in accordance with the range limits of the polarizers. The spatial dimensions varied per target sample, thus the largest data cube had dimensions 500×800×311, corresponding to an 8 cm long tissue sample. The HSI system was integrated into the medical practice and operated by pathology experts (TI and MH). The operator could choose an appropriate region of interest to be imaged and the capture duration was adjusted accordingly. Data capture and postprocessing lasted 2 to 3 min. Additionally, a white reference surface (X-Rite ColorChecker White Balance sheet) and dark signal (lights off state) were imaged with the same system after the capture of the tissue. This resulted in three HSI data cubes per sample, the tissue image $I_{t}$, the white image $I_{w}$, and the dark image $I_{d}$. A black porous material was used as the capture surface, to avoid leaks and contamination of the camera system.

### 2.2 Experimental Dataset

An original HSI dataset was prepared using the custom acquisition system. The experiments in this study were approved by the Ethical Committee of Saitama Medical University (977). All participants gave informed consent for the scientific use of their data. Pigmented skin lesions (PSL) were selected as target pathologies for this study. Lesions that fit the conditions were imaged immediately after surgical excision, before any kind of processing. Tissue samples were imaged with the surface facing up on the capture board. While this process constitutes *ex-vivo* imaging, the fast acquisition (≤30  min after surgery) is analogous to *in-vivo* imaging. Each sample was formalin-fixed and cross-sectioned two days later. The cross-sections were captured both with the HSI system and conventional RGB cameras. The sectioning direction and positioning of cross samples were recorded on the RGB image. Afterward, samples were pressed into paraffin blocks, shaved, and converted into tissue slides, for histopathological evaluation. Next, histological margins were traced on the images of sectioned tissue by expert pathologists (TI and MH). These margins were then traced on the HSI of the raw tissue surface. This resulted in 2D masks of tumor margin that coincided with the spatial dimensions of the HSI. Additionally, a full diagnosis was provided, including disease name and malignancy status (malignant or benign).

The data collection process resulted in the HSI of 32 tissue samples of PSL. Four of the samples were not used for the following reasons. The sample of Paget’s disease had a very different structure compared to the rest of the PSL. Two samples of melanocytic nevus were discarded due to heavy staining with blue dye. A basal cell carcinoma (BCC) sample was discarded due to difficulty in creating the label mask. This resulted in 28 tissue samples from 21 patients. An HSI data cube, a tumor segment mask, disease name, and malignancy status were recovered for each sample. Table 1 shows a detailed breakdown of the dataset. A total of 384,014 spectral signatures were collected for validation, 33% of which were within the tumor margin detected by histology. Another set of 283,206 spectral signatures was collected in the same manner and used for testing. Melanocytic Nevus is closely associated with Malignant Melanoma in terms of structure. These tumors differ from cancer tumors like BCC, due to the absence of keratin in the epithelial layer. Nonetheless, both types of tumor can potentially evolve into malignancy.

**Table 1:** Breakdown of skin tumors included in the dataset.

Diagnosis	Number of samples	Tumor type
Basal cell carcinoma (BCC)	12	Malignant
Malignant melanoma (MM)	1	Malignant
Mucinous carcinoma	1	Malignant
Bowen’s disease	2	Precancer
Melanocytic Nevus	9	Benign
Blue Nevus	1	Benign
Dermatofibroma	1	Benign
Melanophage aggregate	1	Benign

### 2.3 Data Preprocessing

HSI data cubes were preprocessed individually as follows. Normalized reflectance r(λ) for a tissue sample was recovered from each pixel as in

$$r(\lambda )=\frac{I_{t}(\lambda )-I_{b}(\lambda )}{I_{w}(\lambda )-I_{d}(\lambda )},$$ (1)

where $I_{t}(\lambda )$, $I_{w}(\lambda )$, and $I_{b}(\lambda )$ refer to pixel intensities from the target, white reference and dark signal HSI, respectively. To investigate the effect of denoising, preprocessing was followed by denoising with the nonlocal meets global (NGMeet) algorithm.^24^ NGMeet is an iterative algorithm that reconstructs a denoised HSI using both spatial nonlocal similarity and spectral low-rank approximation. Additionally, a mask for the foreground (pixels that correspond to tissue) was created using iterative clustering and morphological operators. Background pixels were set to a flat zero spectrum. For applications per pixel, only the pixels belonging to the foreground mask were used. This resulted in N×311 matrices, where N refers to the number of foreground pixels. Only spectral signatures of the foreground were analyzed because pixel-wise calculations were sensitive to the structure of the background material. For applications per patch, each data cube was split in 32×32×311 patches. Patches that contained no tissue pixels, as well as patches that contained large portions of skin dyes, were discarded. Only the remaining HSI cubes were used for experiments. Augmentation is commonly used as standard practice to improve the training of deep learning models with medical data.^25^ Simple operations such as affine transform are both an obvious choice, as well as easy to implement.^26^ Therefore, the patch dataset was augmented fourfold by vertical, horizontal, and mutual flipping of each channel of the data cube and the labels. Additionally, an algorithm for stitching square patches together was prepared. Thus, after further processing, the resulting square patches could be stitched to recreate the original spatial dimensions of the tissue HSI.

### 2.4 Use Case Scenarios

**Fig. 1:**
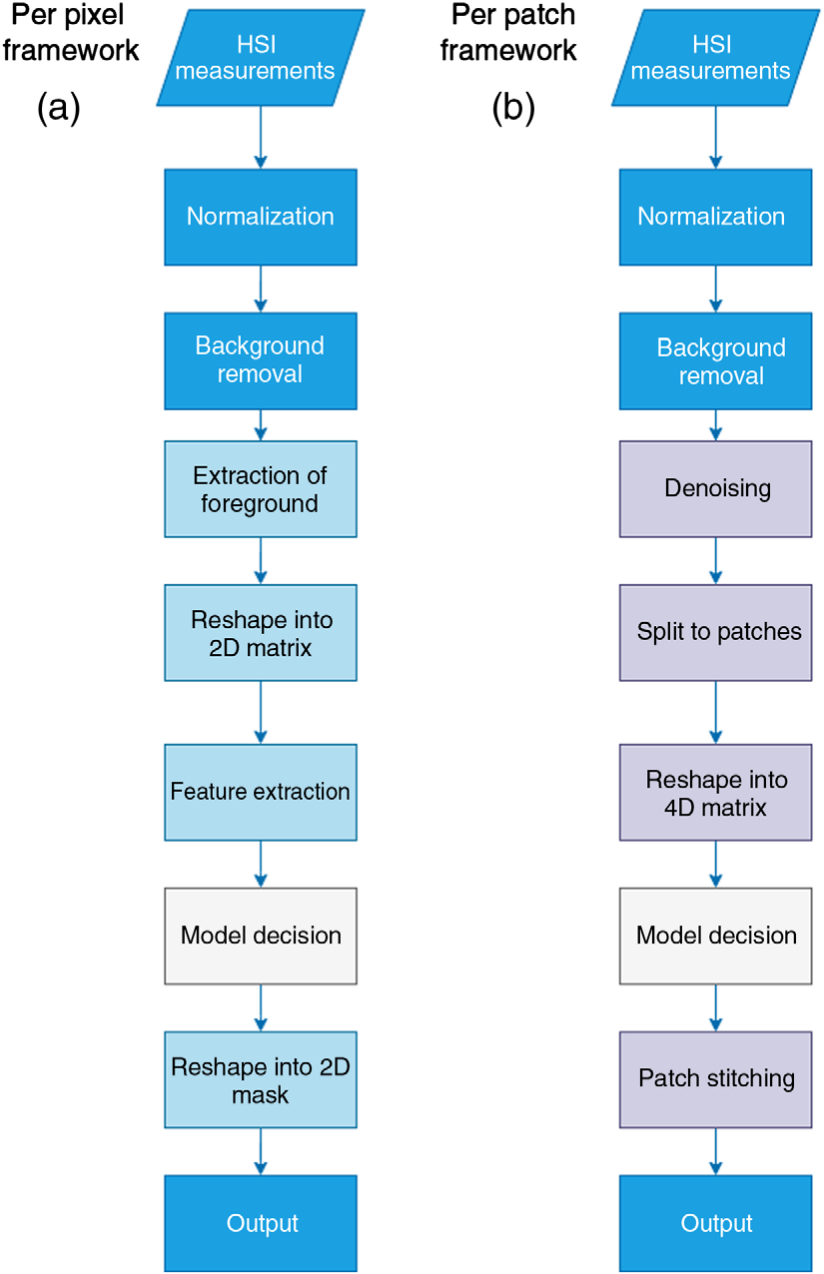
The proposed frameworks for skin tumor segmentation. (a) The pixel-wise framework and (b) patch-based framework have different models at their core. Processes in blocks with vivid blue color are the same for both frameworks.

**Fig. 2:**
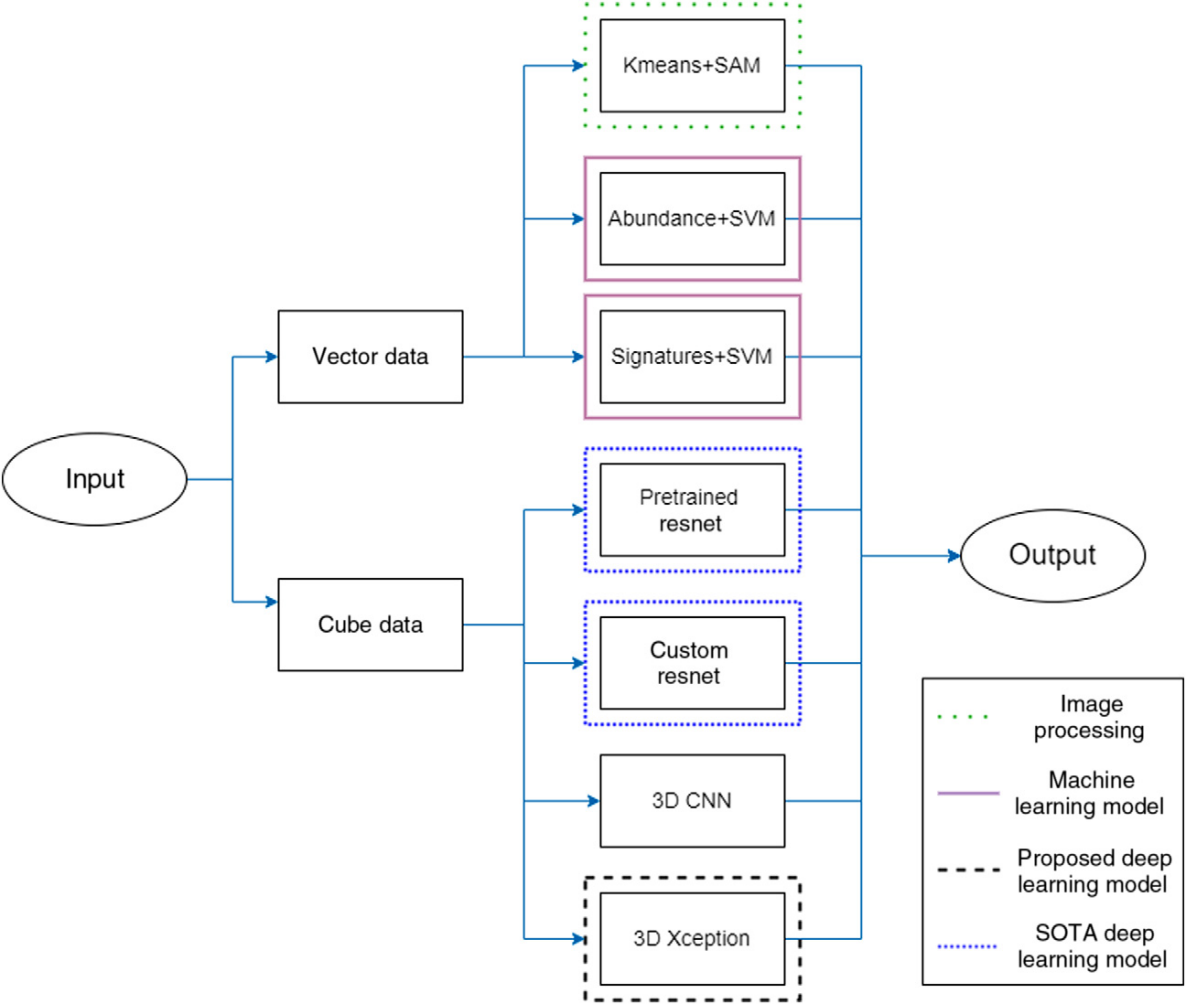
The models that were implemented in this study. Both machine learning and deep learning models are used, each with the respective framework.

Two frameworks were proposed, one for single-pixel processing using machine learning and one for patch-wise processing using deep learning. The reason for the two frameworks is that they are expected to be used in different scenarios. A single-pixel framework can be used when a small number of images/large number of spectral signatures is available and fast system development is required. It can also be used for the initial stages of system development for exploratory analysis. Moreover, a model based on well-defined feature extractors is more easily interpretable and the decision process can be associated with pathophysiological characteristics. On the other hand, previous experiments showed that there is a benefit in using the joint spatio-spectral information of HSI data cubes. Moreover, deep learning classifiers that perform abstract feature extraction have shown exceptional results in applications in medicine. For this reason, the second framework has deep learning at its core. Due to the large size of HSI data cubes and to fit memory and time constraints, a patch-based approach is suggested. This framework is suitable for cases where a large dataset with accurate labels is available. The two frameworks are presented in Fig. 1. The available models are presented in Fig. 2. Each framework is described in detail in the following subsections.

**Fig. 3:**
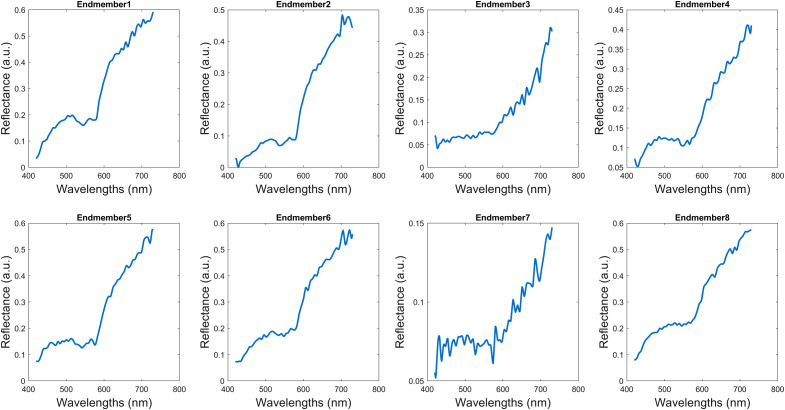
The database of end members used for the calculation of abundance maps.

**Fig. 4:**
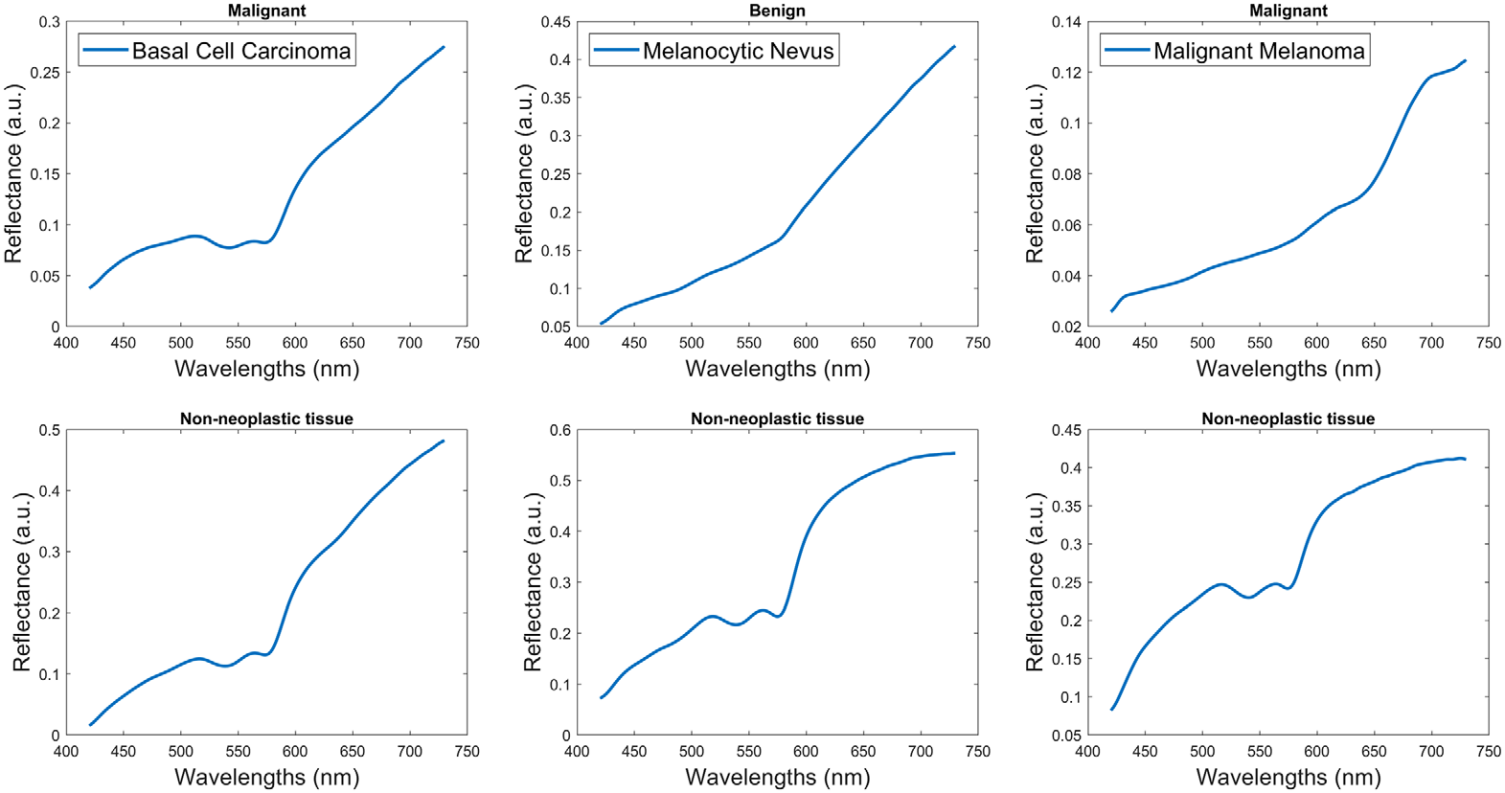
The database of reference signatures used for SAM calculation.

### 2.5 Pixel-wise Framework

The pixel-wise framework in Fig. 1 receives as input the HSI data cube. Pixel-wise normalization is applied and the foreground signatures are extracted based on the foreground mask, after background removal. The signatures from multiple HSI data cubes are reshaped into a 2D matrix, where each row represents the foreground pixels and columns represent the spectral dimension. Nontissue pixels were ignored because their inclusion affects model training. Then, feature extraction is performed. Although there is a wide variety of feature extractors, a simple approach was assessed. Two sets of features were prepared (a) reflectance signatures and (b) abundance maps.^27^ The former refers to the spectral signatures after normalization. Abundance maps were calculated using eight endmembers from a basal cell carcinoma sample. This sample was selected because it contained a variety of colors, textures, and underlying structures. In essence, endmembers represent “pure pixels” or individual substances in the spectral profile of an image target. Endmembers were calculated by the N-Findr algorithm and eight abundance maps were produced, respectively.^28^ The endmembers are shown in Fig. 3. The signature vector was 311-point long, whereas the abundance vector was eight-point long. Then, the feature vectors are fed into a support vector machine (SVM) classifier. This resulted in two schemes: Signature+SVM and Abundance+SVM. On datasets where the two classes have a small amount of overlap and are a bit unbalanced, like this skin HSI dataset, SVM tends to perform well. The SVM model produces a prediction of tumor or normal tissue. The predicted values are reshaped back to the original spatial dimensions of the input images. For comparative purposes, an unsupervised method based on image processing is also implemented. For Kmeans+SAM, the HSI signatures were grouped into seven clusters with Kmeans clustering and then grouped based on SAM similarity.^8^ Six signatures (three of normal tissue and three of PSL tissue) were used as references for SAM calculation, as shown in Fig. 4. MATLAB^®^ 2020b (The MathWorks Inc., Natick, Massachusetts) was used for implementation and experimentation.

### 2.6 Patch-based Framework

For the patch-based framework in Fig. 1, the initial stages of data handling are the same as before. However, in this case, the normalized HSI data cube is split into 32×32×311 patches. Deep models need a lot of memory to work, and HSI data only exacerbate this problem. By splitting data into patches, implementation in reasonable memory levels can be achieved. The patches are reshaped into a 4D matrix of batches, where the dimensions represent (batch number, height, width, and spectrum). The data are fed to a deep-learning model that performs both abstract feature extraction and segmentation. The model produces segmentation results for each patch. The predictions are stitched back together to form the original images.

**Fig. 5:**
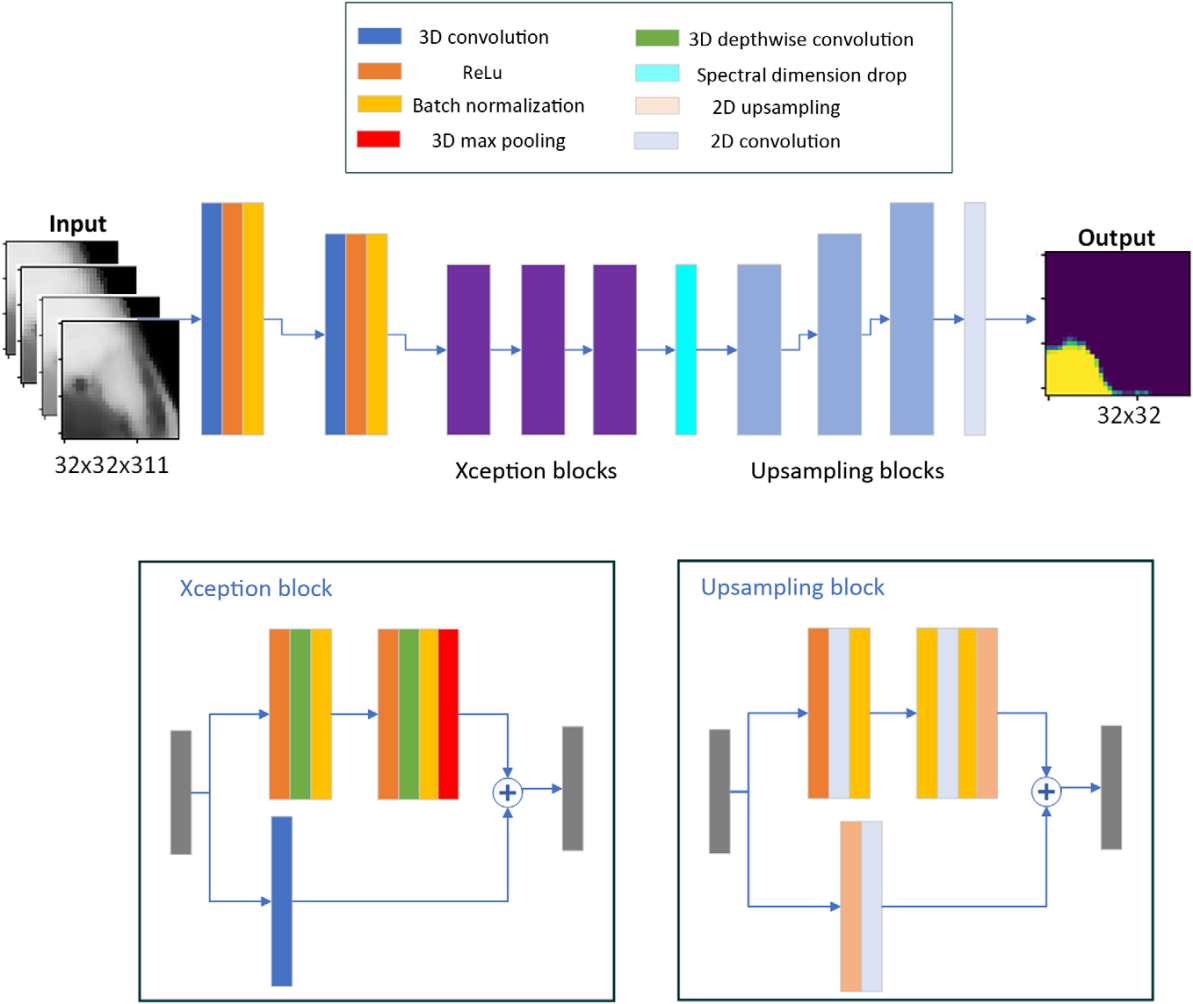
The architecture of the proposed 3D Xception network.

**Fig. 6:**
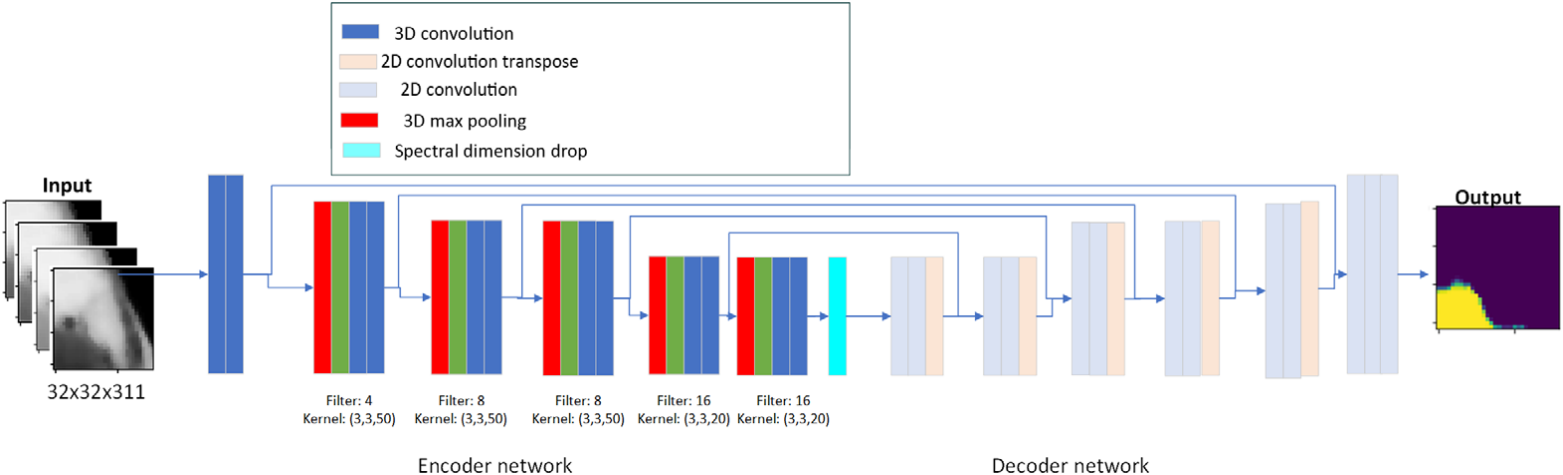
The architecture of the 3D CNN network.

A deep learning model based on 3D convolutions is proposed. 3D Convolution has been used in other segmentation problems in medicine such as MRI diagnosis. The advantage of 3D convolution compared to 2D convolution is the direction of the kernel, which can move along the spectral direction with independent steps. Thus, the kernel for convolution can have different values in the spatial directions and the spectral direction. This way, the information in the spectral direction is preserved and processed individually. The 3D Xception model is based on Xception architecture^29^ with 3D Convolutions instead of 2D. Other deep models, such as Inception need a lot of data to be trained effectively, but Xception can reduce the parameters of the model. Xception architecture outperforms other state-of-the-art (SOTA) segmentation models in problems with natural images. Its feature is that it uses depthwise convolutions instead of common convolutions. Depthwise convolution is performed in two stages to decouple the process. The structure of the proposed 3D Xception is shown in Fig. 5. It preserves the 3D structure in the entire part of the decoder, which enables independent adjustment of the kernel size for convolutions in the spatial and spectral dimensions. A too short kernel size in the spectral dimension could be insufficient to detect patterns in the spectral signatures. At the same time, a too wide size could obscure such patterns. The spectral dimension is dropped at the bottleneck layer. Two different approaches to dropping the 3D at the bottleneck layer were proposed: applying (a) the max function at the spectral dimension or (b) the mean function at the spectral dimension. Dropout layers with a 40% rate were introduced at the end of each Xception block. An additional convolutional neural network based on 3D convolutions (3D CNN) was designed for baseline comparison. The model was structured with a U-Net backbone as shown in Fig. 6, which is commonly used in the medical field for segmentation problems.^13^

**Fig. 7:**
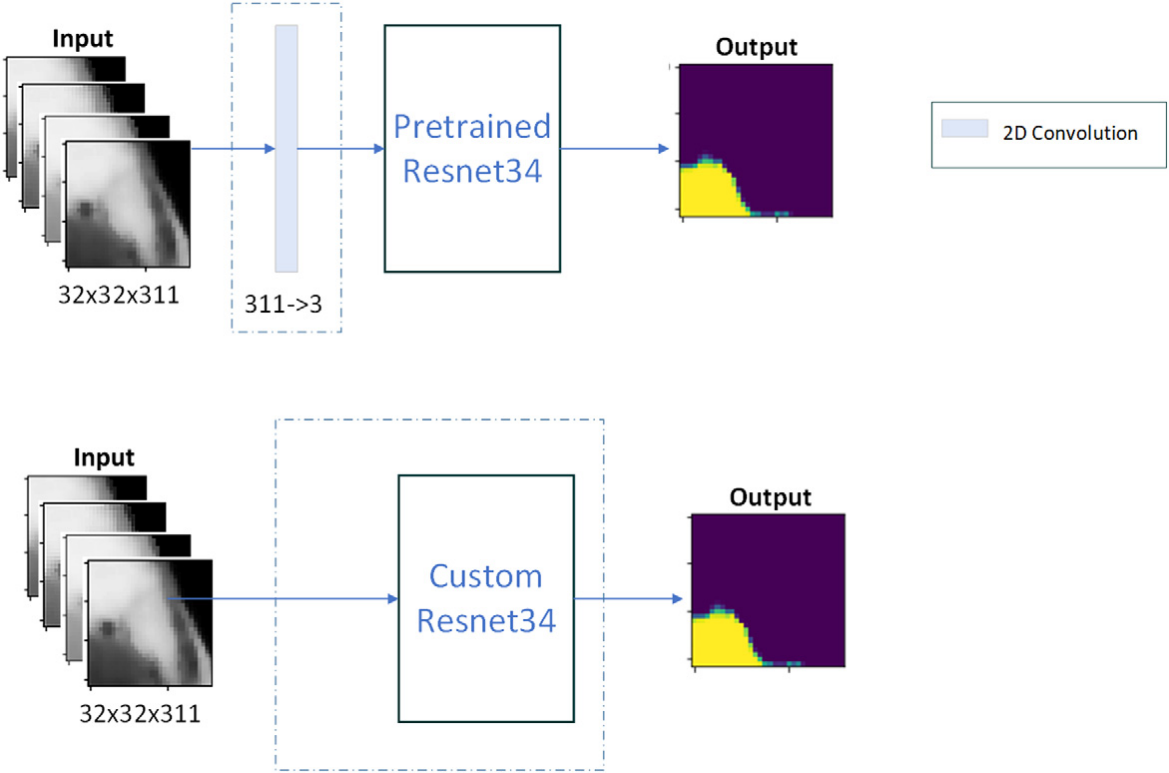
The architecture of the two Resnet-based networks.

The proposed models were compared with various SOTA models. Visual Geometry Group (VGG) and Inception networks showed poor performance in initial experiments, so only the residual network (Resnet) architecture was considered moving forward. A precompiled Resnet34 model^30^^,^^31^ was used in two different configurations, as shown in Fig. 7. The first configuration is pretrained Resnet. Because Resnet is designed to accept three-channel inputs, an additional convolutional layer is added before the input layer to reduce the 311 spectral channels into 3. Only the input convolution weights are trained, whereas the rest of the parameters are frozen to weights trained on the ImageNet dataset. Alternatively, a second configuration is prepared, referred to as custom Resnet. The input dimensions can be passed as a parameter to the model’s input layer, the spectral dimension is reduced with 2D convolutions, and weight training for all layers is performed from scratch. The models were built and tested in an Anaconda (Anaconda, Inc., Austin, Texas) environment, using Python 3.9, Tensorflow 2.7, and a system with NVIDIA RTX6000 GPU.

### 2.7 Training and Evaluation

**Fig. 8:**
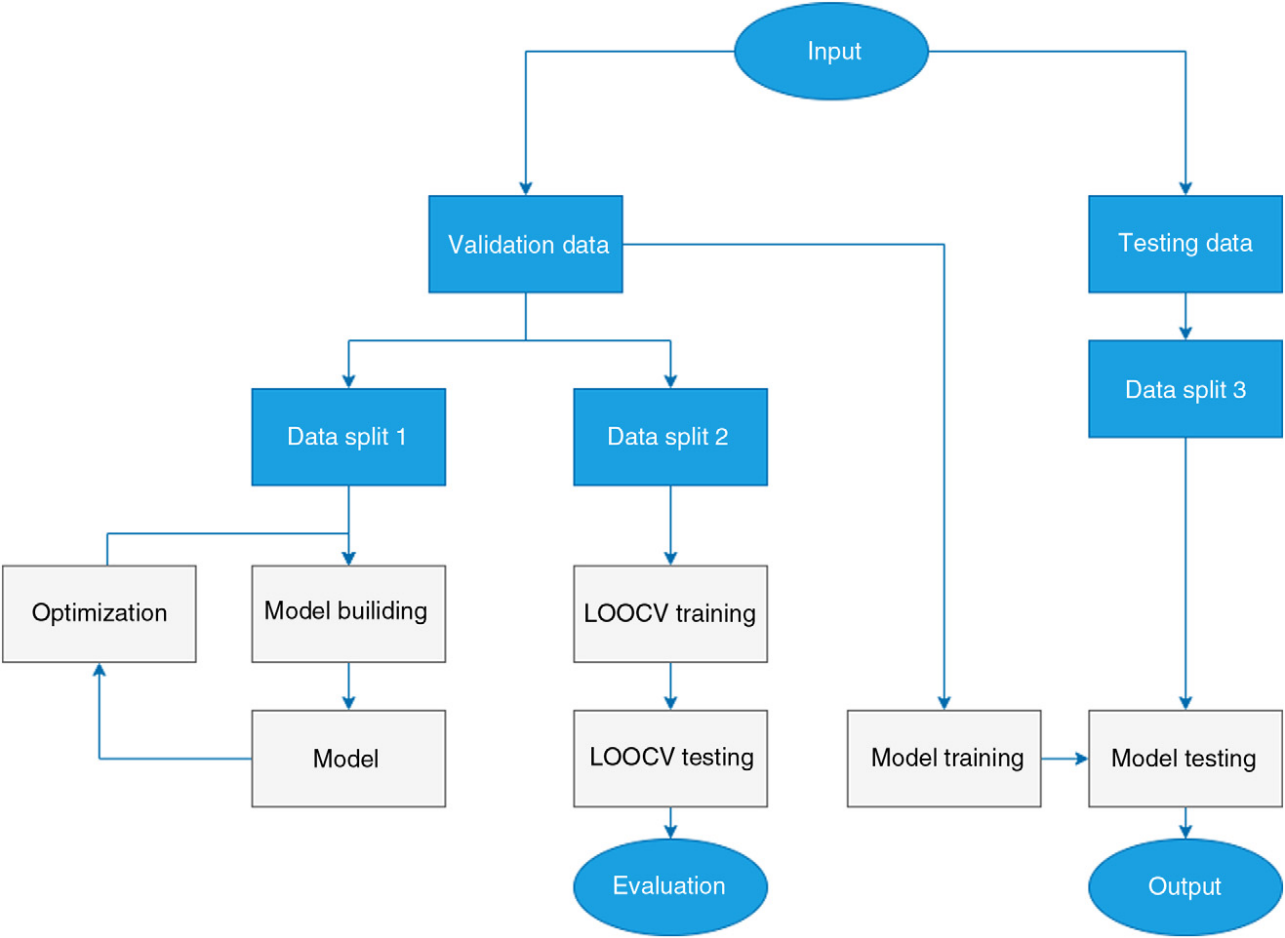
The flowchart of system building, optimization, cross validation, and testing.

Three different data splits were used for experimentation, according to the flowchart in Fig. 8. The input dataset was split into validation and testing datasets. Validation data were split in two different ways. The first data split (data split 1) consisted of 16 training samples and 3 testing samples as described in Table 2. Data split 1 was used for the initial system building and model optimization. Validation data were separated into folds, with each fold consisting of the data derived from a single tissue sample. This structured dataset, data split 2, was used for leave-one-out cross-validation (LOOCV) with 19 folds in total. The training was performed on eighteen folds and performance was evaluated on the last. The training/testing process was repeated until each fold was used for testing once. Average performance was derived from each iteration’s test performance. Finally, the testing data and data split 3 were used for final testing and visual confirmation of the produced segmentation results.

**Table 2:** Data splits.

Split	Usage	Tissue Pathologies
1	Training	Mucinous carcinoma x1, Bowen’s disease x1, BCC x6,
		Melanocytic Nevus x6, Malignant melanoma x1, Dermatofibroma x1
1	Testing	Melanophage aggregate x1, BCC x1, Melanocytic Nevus x1
2	Training	All except one from Mucinous carcinoma x1, Bowen’s disease x1,
		BCC x7, Melanocytic Nevus x7, Malignant melanoma x1,
		Dermatofibroma x1, Melanophage aggregate x1
2	Testing	The unused sample from above
3	Testing	BCC x5, Bowen’s disease x1,
		Melanocytic Nevus x2, Blue Nevus x1

The models in this study were minimally optimized because this is an initial proof of work. The parameters of each SVM (kernel, box constraint, and kernel scale) were optimized with Bayesian optimization. Kernel functions were limited to linear and radial base function (RBF) kernels. Only 10% of available signatures (sampled at equal intervals) were used for training, to avoid a long training duration of the SVM. For the per-patch framework, data augmentation resulted in 1780 HSI cubes for the validation dataset. The deep learning models were optimized using grid search for the following hyperparameters: Optimizer among Adam, RMSProp, learning rate among $10^{-3}$, $10^{-4}$, $10^{-5}$, $10^{-6}$, exponential decay among none, $10^{-5}$, $10^{-6}$, loss function among binary cross entropy (BCE), binary cross entropy+Jaccard coefficient loss (BCE+JC). BCE is the most common loss for segmentation problems. However, in cases where the classes are unbalanced, model training converges at a good accuracy for the prevalent class, instead of focusing on both classes. To alleviate this problem, JC loss has been proposed.^17^^,^^32^ The losses are defined using the predicted value PR and the ground truth value GT as follows:

BCE(PR,GT)=−GT·log(PR)−(1−GT)·log(1−PR), (2)

$$\mathrm{JCL}(\mathrm{PR},\mathrm{GT})=1-\frac{\mathrm{PR}\cap \mathrm{GT}}{\mathrm{PR}\cup \mathrm{GT}},$$ (3)

BCE+JC(PR,GT)=BCE(PR,GT)+JCL(PR,GT). (4)

Both PR and GT are 2D binary masks of the segmentation result. Two alternative structures were investigated for the 3D Xception network. The first consisted of four blocks with filters at [64, 128, 256, 728] and the second of three blocks with filters [64, 128, 256]. While the kernel size of 3D convolution was kept at three pixels for the spatial dimensions, values [5, 10, 20, 30] nm were investigated for the spectral dimension.

Accuracy, sensitivity, and specificity metrics were used for evaluation. These metrics are commonly used in classification problems but are lacking when evaluating performance concurrently in the two spatial dimensions. Accuracy expresses the ratio of correct prediction. Sensitivity expresses the ratio of positive cases being detected. Specificity expresses the ratio of a negative prediction being correct. Sensitivity and specificity are commonly compromised with each other because in a medical setting no positive case should be missed, but high false positives induce an unnecessary burden to the patient. Additionally, Jaccard coefficient (JC), also known as intersection-over-union was used as the main performance metric. JC is more appropriate for segmentation tasks because its calculation is unaffected by a small number of true positives or true negatives. The performance metrics are calculated as:

$$\text{Accuracy}=\frac{\mathrm{TP}+\mathrm{TN}}{\mathrm{TP}+\mathrm{FP}+\mathrm{TN}+\mathrm{FN}},$$ (5)

$$\text{Sensitivity}=\frac{\mathrm{TP}}{\mathrm{TP}+\mathrm{FN}},$$ (6)

$$\mathrm{Specificity}=\frac{\mathrm{TN}}{\mathrm{TN}+\mathrm{FP}},$$ (7)

$$\mathrm{JC}=\frac{\mathrm{PR}\cap \mathrm{GT}}{\mathrm{PR}\cup \mathrm{GT}}=\frac{\mathrm{TP}}{\mathrm{TP}+\mathrm{FP}+\mathrm{FN}},$$ (8)

where TP, TN, FP, FN, stand for true positive, true negative, false positive, and false negative prediction, respectively. PR stands for predicted value and GT for ground truth. All values can be expressed as percentages. Moreover, the balance between sensitivity and specificity was evaluated with the receiver operator characteristics (ROC) area under the curve (AUC), which is commonly used for classification and segmentation models.^33^ The average and standard deviation of the evaluation metrics are reported for LOOCV.

## 3 Results

### 3.1 System Building

**Fig. 9:**
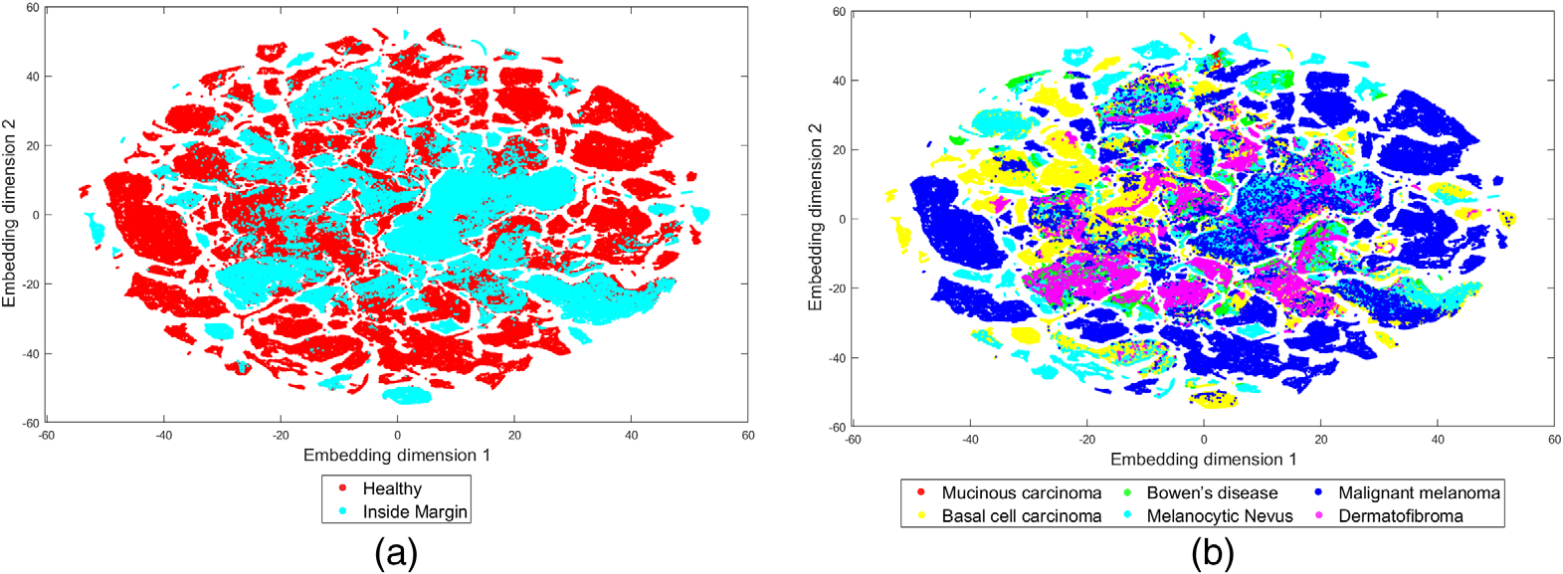
t-SNE visualizations of the dataset (a) according to tumor status and (b) according to PSL type.

Due to the high-dimensionality of the HSI data space, the validation dataset is visualized using t-distributed stochastic neighbor embedding (t-SNE). This technique uses a distance metric to reduce each spectral signature to a 2D or 3D embedding, and it is well suited for high-dimensional spaces. Two-dimensional embeddings and euclidean distance were used in this case. The embeddings of the labeled dataset are displayed in Fig. 9. The two-dimensional embedding shows a great overlap among pathologies, tumor tissue and non-neoplastic tissue. This fact highlights the difficulty of the problem of tumor segmentation, which requires a nontrivial solution.

**Fig. 10:**
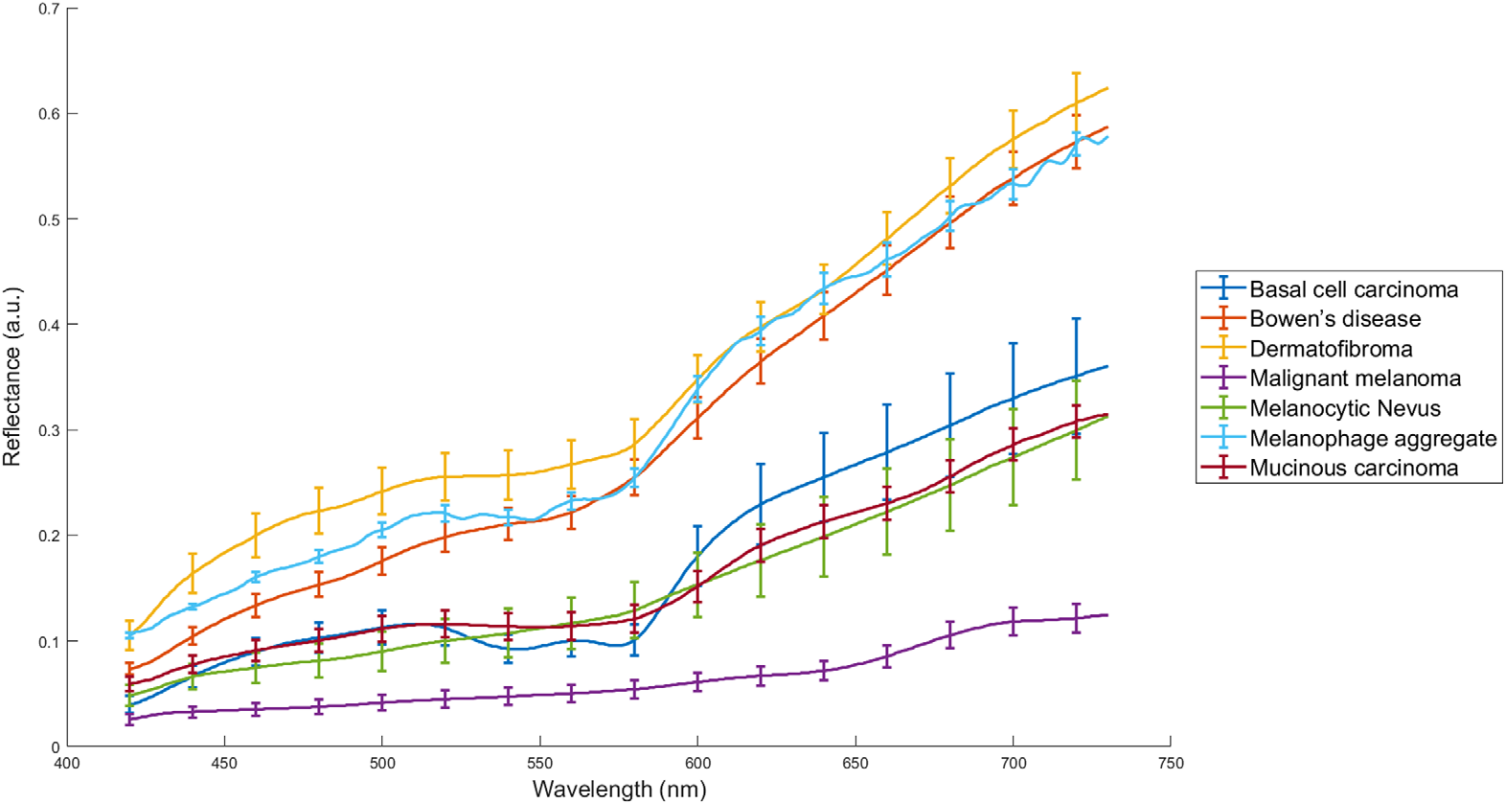
Average signatures for different pathologies. The error bars represent 20% of the standard deviation across signatures for each pathology.

The average signature for each pathology in the validation dataset is presented in Fig. 10. Average signatures show a clear difference among pathologies, both in curve shape and curve amplitude. Melanocytic nevus and Melanoma show a straight appearance and a sudden upward slope around 600 and 650 nm, respectively. Basal cell carcinoma shows a distinct double valley at 540 and 570 nm, but at a much lower reflectance compared with benign pathologies. Additionally, Basal cell carcinoma and melanocytic nevus show a much higher standard deviation compared to other pathologies. However, this fact could be attributed to the large number of available tissue samples and the signatures for those two categories.

Each model was optimized using the train/test splits of data split 1. The SVM model was optimized using Bayesian optimization at 200 evaluations. This resulted in observed parameters 44.184 for box constraint and 4.0136 for the kernel scale of the RBF kernel. For Kmeans+SAM, values proposed in the literature were used. Specifically, signatures were split into seven clusters and the sum of SAM similarity per pixel in the cluster was used for segmentation.

**Fig. 11:**
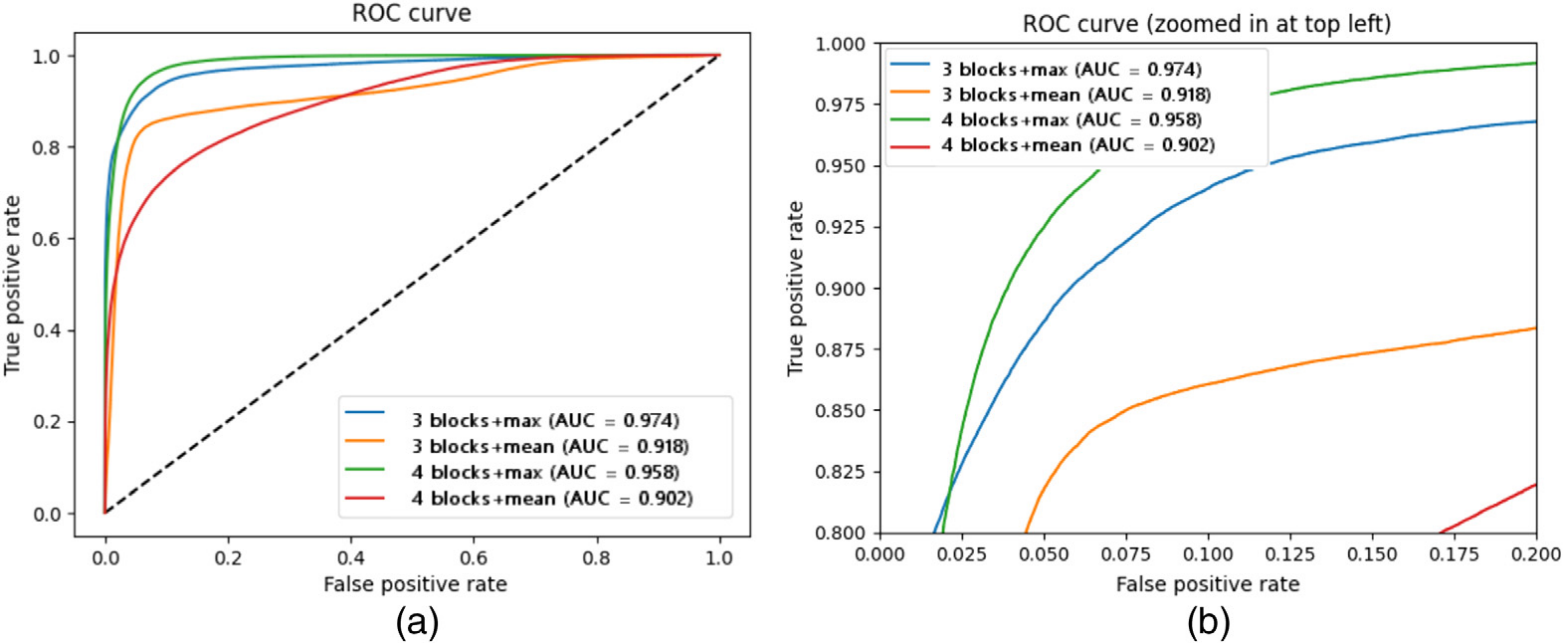
The influence of the structure of the 3D Xception network on the AUC performance. (a) In-full view, and (b) zoomed-in at the knee of the curve.

Four alternative structures were investigated for the 3D Xception network. The balance of sensitivity and specificity in terms of AUC for the different structures is shown in Fig. 11. Networks with max function at the bottleneck layer display higher AUC compared with the alternative. Initially, four Xception blocks show improved AUC performance compared with three blocks. The convergence of the training process at 200 epochs is also affected. High loss performance during training and testing for the four-block network shows that it suffers from overfitting. The three-block network has lower training and testing loss, as well as smoother convergence. Therefore, a network with three Xception blocks is used in subsequent experiments.

**Fig. 12:**
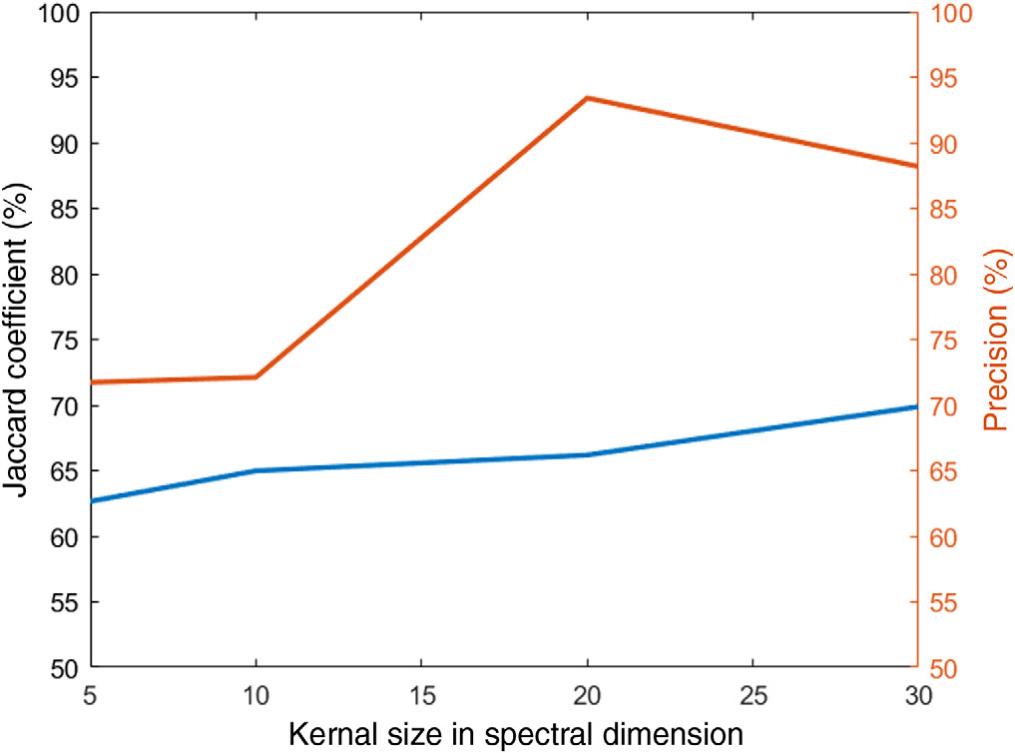
The influence of kernel size at the spectral dimension in testing JC and precision.

The influence of kernel size in the spectral dimension on testing performance is shown in Fig. 12. While JC increases as the kernel size increases, this comes at a cost of precision at 30 nm. After further experimentation, the kernel (3, 3, 10) was used for the first block and (3, 3, 20) for the last two blocks of the 3D Xception network. Dropout layers with a dropout rate 40% were also applied at the end of each block, to avoid overfitting during training. Furthermore, the choice of training parameters greatly affects model performance, as expected. Training with BCE+JC loss converged more smoothly and at a lower value compared to training only with BCE loss. The combination of RMSProp with BCE+JC loss achieved the best performance for all deep learning models. The learning rate was optimized at 0.0001 for ResNet, at 0.001 for 3D CNN, and at 0.00001 for 3D Xception.

### 3.2 Validation Performance

LOOCV was performed twice with the same model parameters for both the preprocessed and the denoised data. This way the effectiveness of spatial denoising with the NGMeet algorithm can be evaluated. Performance metrics without denoising and with denoising are provided in Tables 3 and 4, respectively. Accuracy and specificity metrics paint a misleading picture about the overall performance of segmentation, due to the high prevalence of the negative class. For this reason, more emphasis should be given to JC, AUC, and sensitivity metrics. Out of all the metrics, JC performance is deemed as the most important, due to the balance it provides between false positives and false negatives. Denoising did not improve and even slightly reduced the performance for pixel-wise models. JC performance of preprocessed data without denoising came with increased sensitivity and slightly increased specificity for Abundance+SVM and Signature+SVM. On the other hand, spatial denoising improved JC performance for patch-based models. However, JC performance improved at a cost of specificity.

**Table 3:** Cross validated performance with LOOCV (without denoising).

Method	Accuracy (%)	Sensitivity (%)	Specificity (%)	JC (%)	AUC
Kmeans	**89.67**	51.46	**93.70**	33.64	0.726
Abundance+SVM	78.05	**66.03**	79.33	47.89	0.960
Signature+SVM	80.30	64.69	86.26	**50.17**	**0.996**
Pretrained Resnet	87.24	**68.88**	60.62	40.99	0.829
Custom-Resnet	87.59	65.71	63.09	**42.68**	0.859
3D CNN	78.22	46.25	**84.19**	33.59	0.845
3D Xception	**87.91**	61.54	68.70	42.12	**0.897**

Note: Values are reported as an average across all folds. The best result for each metric per framework is highlighted in bold font.

**Table 4:** Cross validated performance with LOOCV (with denoising).

Method	Accuracy (%)	Sensitivity (%)	Specificity (%)	JC (%)	AUC
Kmeans	**89.75**	52.49	**93.52**	34.88	0.730
Abundance+SVM	79.50	52.66	92.03	43.92	0.990
Signature+SVM	79.26	**61.17**	86.15	**47.53**	**0.991**
Pretrained-Resnet	**88.81**	**71.62**	66.32	**45.87**	0.881
Custom-Resnet	87.13	67.59	59.04	43.12	0.850
3D CNN	80.26	48.17	**86.58**	33.92	0.887
3D Xception	88.28	63.43	68.15	43.03	**0.914**

Note: Values are reported as an average across all folds. The best result for each metric per framework is highlighted in bold font.

**Fig. 13:**
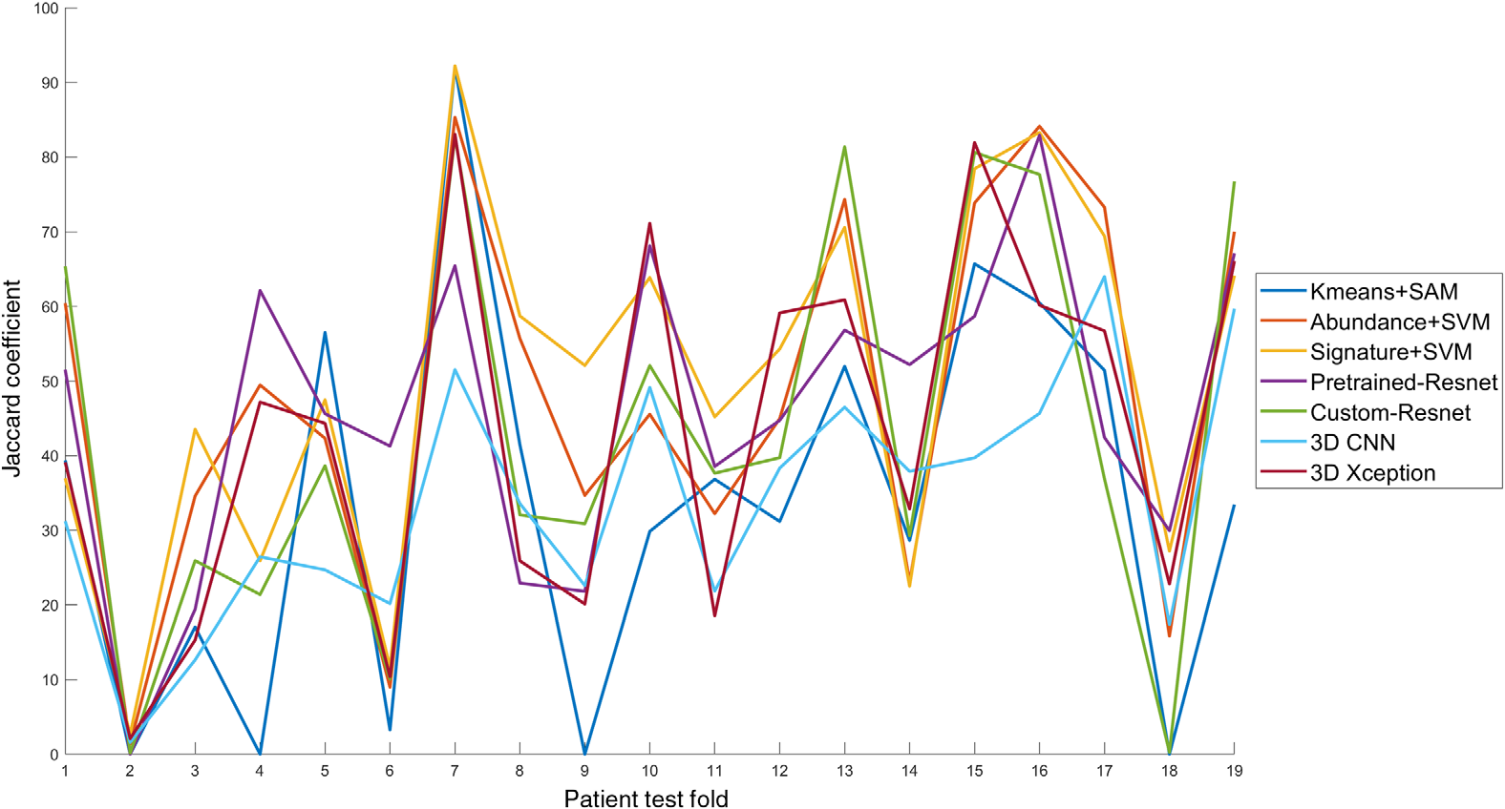
Performance in terms of Jaccard coefficient per fold.

The fluctuation of JC across folds is presented in Fig. 13. For pixel-wise models, preprocessed data is used, whereas patch-based models were trained and tested with denoised data. A tissue sample that was diagnosed as Melanophage aggregate (benign neoplasia), two BCC, and a melanocytic nevus had the worst prediction in terms of JC. It needs to be emphasized that the ground truth labels are approximate because they refer to the histological evaluation of specific cross-sections on the tissue. For the Melanophage Aggregate sample, since the true positive area is just a small growth of melanocytic cells, it is difficult to detect. SVM models detected both the true positive and another false positive area on the left. Pretrained-Resnet detected only the false positive. This false positive area was not evaluated by a cross-section. Therefore, there is a chance that the false positive was a true positive that remained undetected due to the benignity of the diagnosis. The BCC test sample at fold 6 has an unusual appearance where the epidermal layer of the tumor tissue was shed and the deeper cells are prominent. Almost all models missed its detection apart from pretrained-Resnet, which detected almost the entire tissue sample as tumorous. For the other BCC sample (fold 9) where models failed, the mapping of histological ground truth was not successful. The same holds for the Melanocytic Nevus at fold 18. This may affect the metric calculation and should be assigned lower importance during model evaluation.

**Fig. 14:**
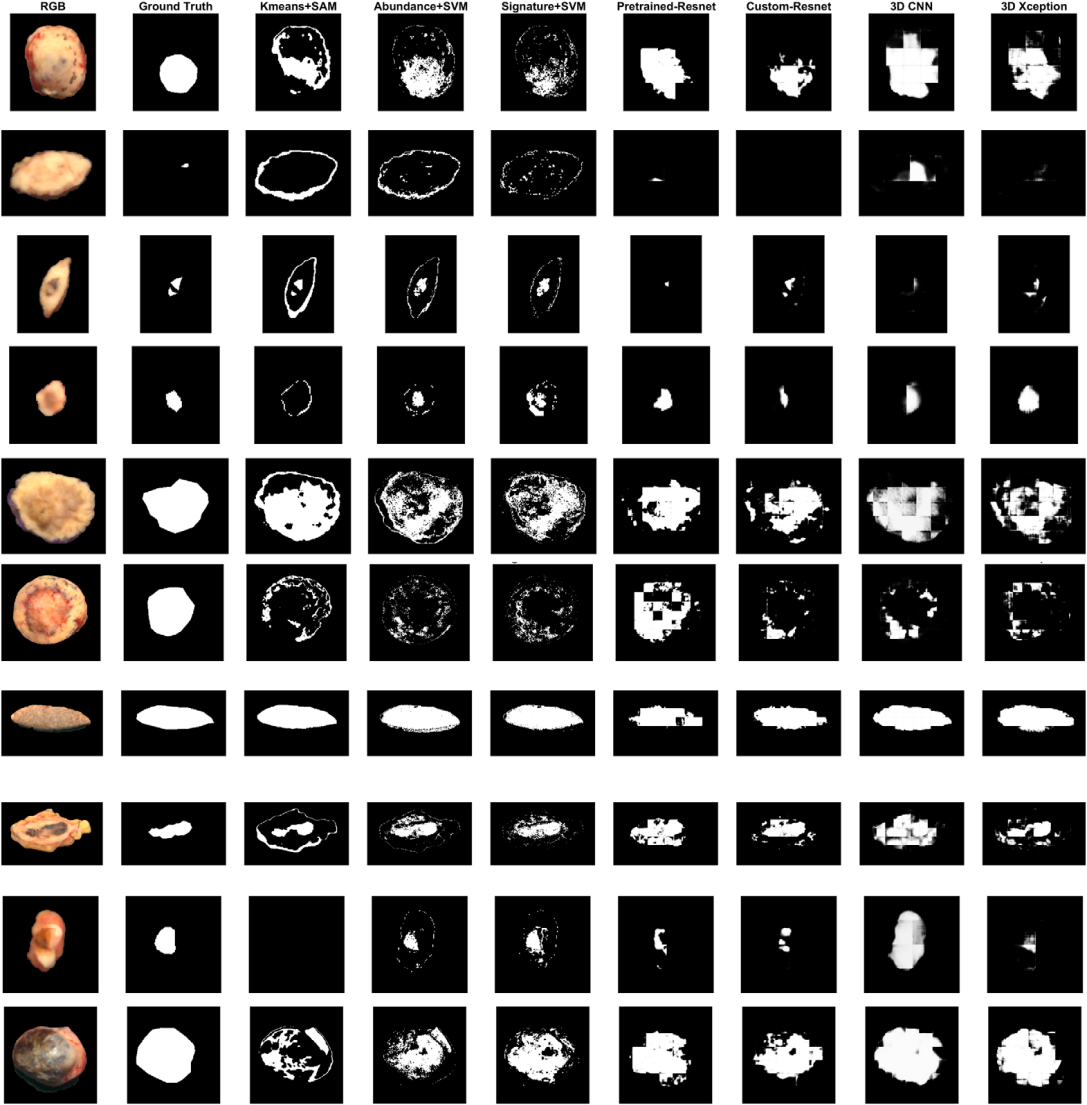
LOOCV segmentation results for folds 1 to 10. From top to bottom: Mucinous Carcinoma, Melanophage aggregate, BCC, BCC, Bowen’s disease, BCC, Melanocytic Nevus, BCC, Melanocytic Nevus, BCC.

**Fig. 15:**
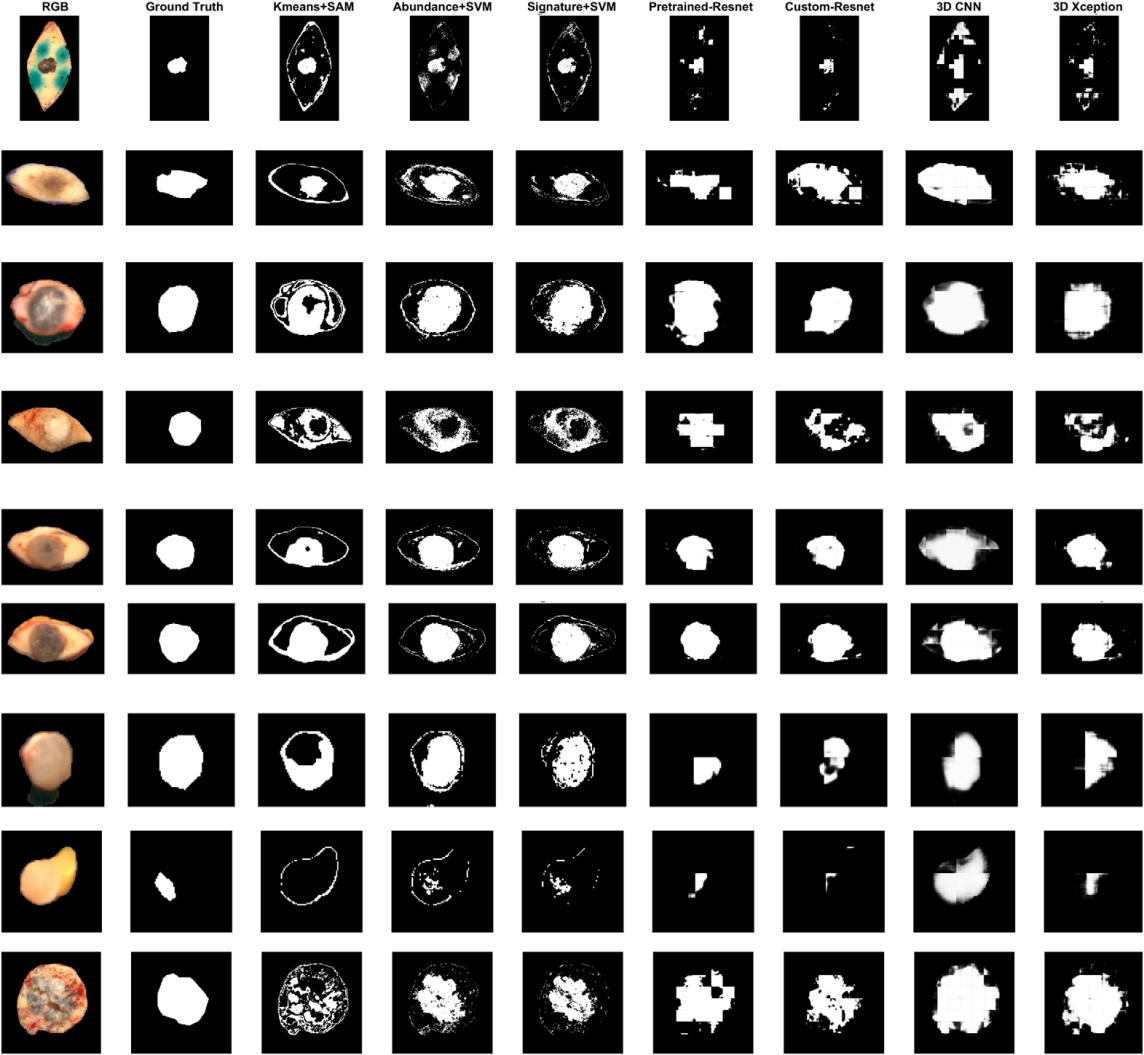
LOOCV segmentation results for folds 11 to 19. From top to bottom: Melanoma, Melanocytic Nevus, BCC, Dermatofibroma, Melanocytic Nevus, Melanocytic Nevus, Melanocytic Nevus, Melanocytic Nevus, Melanocytic Nevus, BCC.

The segmented masks at each test sample of 19 folds of the LOOCV are provided in Figs. 14 and 15. The preprocessed dataset without denoising was used for Kmeans+SAM, Abundance+SVM and Signature+SVM. The denoised dataset was used to train the patch-based models. All pixel-wise models suffered from false positive detection at tissue boundaries. Patch-based models did not suffer from such a problem. This effect was even more pronounced for Kmeans+SAM. Moreover, Kmeans+SAM failed to detect any tumor at all in four cases. Kmeans+SAM suffers from detecting illumination artifacts and areas with high contrast from the surroundings, instead of detecting tumor growths. This is especially evident in folds 1, 8, 10, 13, 14, and 15. Abundance+SVM detected more homogeneous tumor masks compared to Signature+SVM. However, it suffered from increased false positives, for example in the cases of BCC (fold 10) and melanoma (fold 11).

Patch-based models provided more compact and dense masks compared to pixel-wise models. Dense masks are following the biological structure of the tissue. However, they all suffered from false positives and overestimation of the tumor area. Pretrained-Resnet performed better in terms of JC compared to custom-Resnet. Pretrained-Resnet was the only model that detected tumor at the center of the BCC sample at fold 6. However, both it produced much larger tumor masks compared with custom-Resnet, showing a tendency of high false positives. Additionally, pretrained-Resnet tended to label an entire patch as only tumor or only non-neoplasia, resulting in square-shaped segments. This fact is discordant with the shape of tumor borders, which tends to be circular or wavy. 3D Xception produced more curvy tumor segments, although not as dense. False positives were more controlled compared to Pretrained-Resnet. However, the 3D Xception model falsely detected a non-neoplastic edge at the melanoma sample, with more emphasis compared with the Resnet models. This could be attributed to the influence of staining, which was reduced but not completely eradicated by removing the more heavily stained patches. 3D CNN fails to detect the tumor area and mostly detects tissue pixels against the background.

### 3.3 Testing Performance

**Fig. 16:**
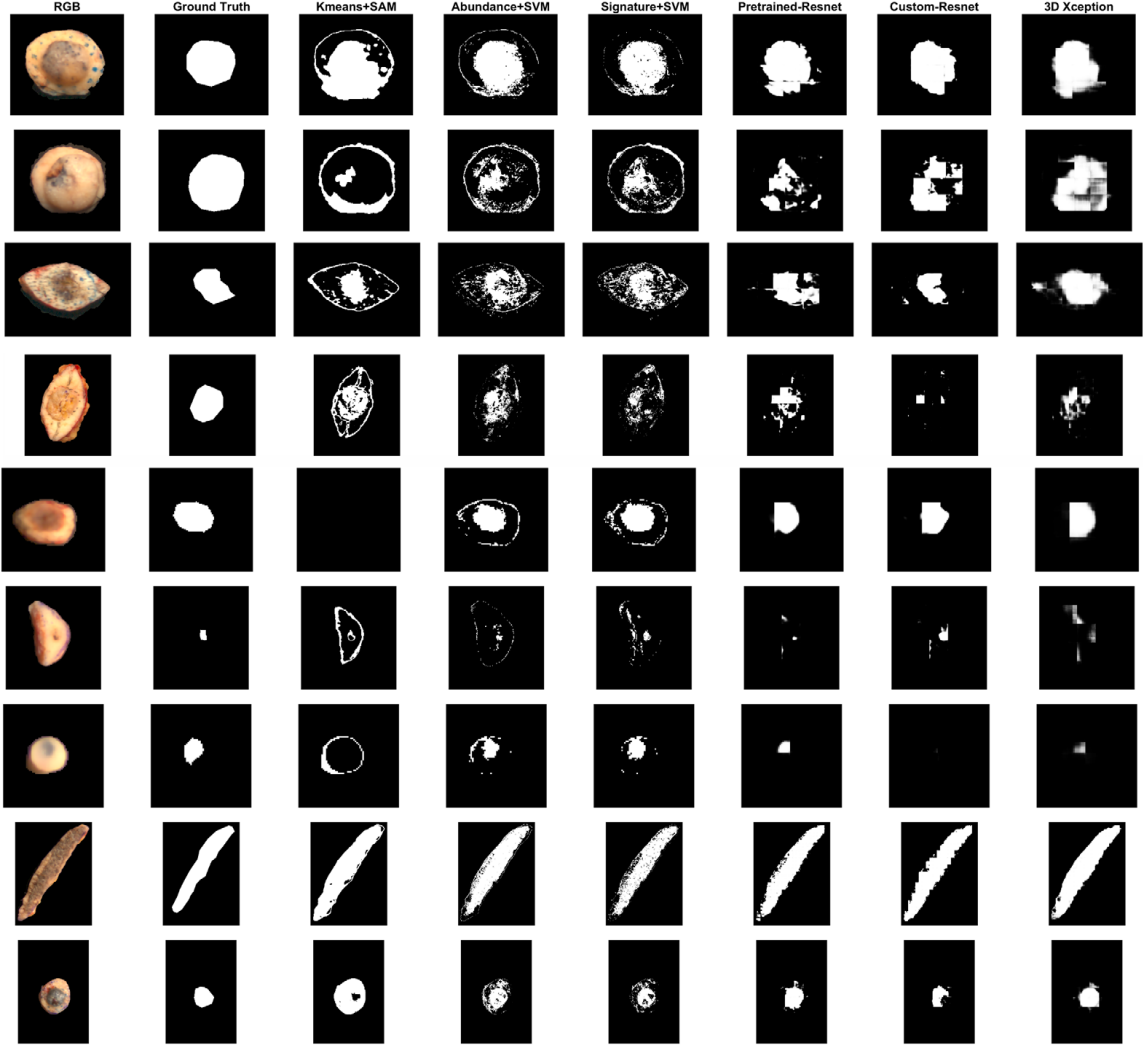
Segmentation results for test samples 1 to 9. From top to bottom: BCC, BCC, BCC, Bowen’s disease, Melanocytic Nevus, BCC, Blue Nevus, Melanocytic Nevus, BCC.

After the validation stage, the models are trained on the entire validation dataset and tested on an unknown testing dataset, data split 3. Segmentation results for each sample are demonstrated in Fig. 16. 3D CNN results are not included during testing, because it was falsely detecting skin instead of the tumor during validation. It should be noted that the test dataset contained one blue Nevus sample, a pathology that was not present in the validation and training dataset. Performance fluctuates a lot depending on the pathology of the test sample. Performance is exceptionally good for melanocytic PSL. The issue of SVM detecting tissue edges as false positives is still present. In terms of technical evaluation, deep learning models provide clearer tumor masks for BCC samples compared to SVM models, with fewer false positives in non-neoplastic areas. However, in terms of medical evaluation, SVM-based pixel-wise masks are more appropriate in cases where tumor detection was successful. Although margins are not delineated well and false positives appear in areas with non-neoplastic tissue, all models apart from Kmeans+SAM detect the general area of the tumor.

LOOCV and testing results were examined by two expert pathologists (TI and MH). The consensus was that the tumor masks were still not sufficient for computer-assisted diagnosis and further improvements are needed. SVM-based models were deemed as closer to the expected ground truth, despite the false positives around the tissue border.

## 4 Discussion

In this study, a new HSI dataset of various PSL was created. The average spectral signatures for different pathologies in Fig. 10 are in agreement with the observations by Borisova et al.,^34^ who found that pathologies with high vascularization or high melanin content have distinct signatures. Considering that tumor growths are associated with larger vascular networks and increased melanin content in deeper dermal layers, the shape of the recovered signatures is pathophysiologically sound. When the number of pathologies becomes nontrivial with the introduction of more than the dipole of nevus and melanoma, tumor detection and classification become more difficult. Therefore, simple approaches based on thresholding or clustering are no longer sufficient.

The experiments in the present study showed that margin detection of skin PSL using HSI data is feasible. All models managed to detect the general area of the tumor. Pixel-wise classification suffers from false positives in the non-neoplastic region of the tissue, far from the tumor area (false detection of edges, shadows). This can be improved with post-processing using morphological operators. However, an adaptive post-processing scheme is preferred, for small tumor areas (e.g., the melanophage aggregate sample) to be preserved. NGMeet denoising does not improve results and even reduces performance. Since decision-making is based on individual pixels rather than spatiospectral patterns, spatial denoising is expected not to be of assistance. The decrease in performance can be attributed to useful spectral components, which were discarded at the SVD reconstruction step of the denoising algorithm. Kmeans+SAM was the only method that failed to detect a tumor region in more than one case, indicating a high false negative rate. Additionally, it consistently misclassified shades and borders, in a much higher degree compared to SVM-based methods. Additionally, Kmeans+SAM detects artifacts and areas with different illumination distribution, instead of tissue components. SVM-based models were affected much less by such problems. However, they still suffered from nondense masks and detection of tissue borders as false positives.

The quantitative results of this study showed similar performance for the different methods. SVM, a simple model, can provide relatively good results. However, SVM is difficult to scale and update for a large number of know data and pathologies. The most simple method, based on Kmeans, failed in detecting a tumor area in some cases, so it is inappropriate. In terms of scalability, standardized implementation and access to decision parameters, more complex methods such are deep learning methods have potential. Deep learning models use convolutions to extract spatio-spectral features. They can prepare more comprehensive tumor masks compared to SVM models, by simultaneously evaluating all dimensions. There is sufficient room for improvement with deeper networks or larger datasets. Pretrained-Resnet had the best performance in terms of JC, but returned a lot of single-class predictions per patch, indicated by the straight edges on the masks. 3D Xception achieved similar performance, and at the same time, it followed the shape of the tumor more appropriately. The size of the convolution kernel can be adjusted in 3D Xception network, which is not possible for ResNet with 2D convolutions. Therefore, 3D Xception has more potential for improvement with detailed optimization of the structure, hyperparameters, and training process.

Although the segmentation problem for PSL using HSI data is a relatively new problem in literature, relevant studies are mentioned in Table 5. As mentioned before, accuracy and specificity are not appropriate metrics for an unbalanced dataset, therefore visual confirmation and JC are more useful for evaluation. The performance in the present study is similar to other works, however, none is sufficient for clinical application yet. Leon et al.^8^ achieved good JC performance with Kmeans+SAM, but the results were validated on a single fold and tested on another. No cross validation was performed. In two out of the 10 test samples, no lesion region was segmented at all. Additionally, mostly flat, evenly textured samples of melanocytic lesions were included in the dataset. The dataset lacked the variety in pathologies that were present in the dataset of this study. The ground truth was not derived from histology but was produced experimentally with a semiautomatic labeling tool using five *in-situ* signatures of healthy and malignant tissue. Moreover, the dataset of Leon et al.^8^ included additional depth information in the near infrared (NIR) range, which was not available by our custom acquisition system. The spectral resolution was 8nm, much lower compared to the 1nm resolution in the present study. Halicek et al.^35^ performed segmentation on head and neck samples, including oral and gland tissue, but no skin tissue. They achieved good results with an Inception CNN with adjusted input dimension, similar to the custom-Resnet of the present study. However, a key difference exists in the training and evaluation process. Histology slides from surface sections were prepared instead of cross sections. Surface sections are not available in standard pathology practice, which hinders the applicability and update of such a framework. Moreover, pixels close to the tumor border were not included in the metric calculation. In this case, as well, the NIR range was included in the processing. Their HSI acquisition system was capable of 5-nm spectral resolution. Zunair et al.^36^ improved the segmentation of tuberculosis from CT scans using 3D convolutions. Xu et al.^17^ proposed the use of a logarithmic JC loss for the improvement of Xception-based segmentation during remote sensing. Both 3D convolutions and JC loss were used in the present study, with positive effects on performance.

**Table 5:** Performance of other models in literature.

Method	Accuracy (%)	Sensitivity (%)	JC (%)	AUC
Clustering+SAM ^8^	N/A	N/A	57.93 (13.53)	N/A
*In-situ* PSL, VIS+NIR range				
Inception CNN^35^	92 (9)	92 (8)	N/A	0.95
*Ex-vivo* thyroid, VIS+NIR range				
Inception CNN^35^	81 (11)	81 (15)	N/A	0.82
*Ex-vivo* oral, VIS+NIR range				

Note: Metrics are reported as average accompanied by the standard deviation in parenthesis. Row 1 metrics are validated at a single fold. Rows 2 to 3 show metrics validated with LOOCV.

Despite the improvements and additional insights compared to previous works, this study has considerable limitations. The process of ground truth recovery included multiple approximation steps. The ground truth masks were drawn manually on images of the cross sections and then traced to images of the surface view. These two manual steps increase the cases where a spectral signature and a label are mismatched. Moreover, the tracing itself depends solely on the cross-section labels. This means that there is a chance of false negatives existing on the label, due to those areas not being part of the cross-section. Consequently, the ground truth is approximate and not absolute. This affects both training and evaluation performance. From the perspective of pathologists, a low false positive rate is more important than high sensitivity. However, overt focus on low false positives during training can hinder effective training. The dataset contained a large number of BCC and melanocytic nevus samples, but only one melanoma. The diagnostic performance of dermatologists on BCC detection is exceptionally high, compared with other pathologies. However, there is merit in this study, because nondermatologist medical personnel is commonly asked to evaluate tissue. An HSI-based tumor detection tool could assist nondermatologist personnel to an even greater degree. Practical pathology knowledge, e.g., that melanoma appears redder compared to nevus due to high hemoglobin content, was not explicitly used for the models in this study. Optimization of the models in synergy with experienced doctors could further improve performance. Further investigation into training and model structure is necessary for the proposed frameworks to be helpful in a clinical setting.

This study proposed two new HSI-based frameworks and the first work to our knowledge, which focuses on tumor segmentation of PSL. A lot of directions can be followed in terms of future improvements until implementation in clinical practice is viable. While the SVM model showed the best performance, SVM has limitations regarding scalability when the available data number increases. However, pixel-wise segmentation could be improved with a more targeted training dataset and adaptive post-processing. Deep learning models have a large room for improvement with detailed optimization of the model structure and experimentation with the size of the convolutional kernels. Alternative metrics, further focused on skin tumor segmentation could improve training performance and improve generalization. Common loss functions and evaluation metrics do not accurately describe performance in accordance with the view of medical experts. Therefore, further work is necessary to generate metrics that are appropriate for the evaluation of tumor segments. Finally, feature visualization techniques and interpretation of HSI decision-making using explainable artificial intelligence (XAI) are required before practical implementation.

## 5 Conclusions

Tumor segmentation of HSI data is a field in gross pathology. This work proposed two frameworks, a pixel-wise and a patch-based framework for the segmentation of HSI data from PSL. Results showed that SVM-based models can achieve good JC performance but produce noisy tumor masks. Joint inclusion of spatio-spectral information by deep learning models produces more comprehensive tumor segments. Xception networks with 3D Convolution can achieve performance similar to SOTA models while allowing for further customization in terms of convolution in the spectral direction. Good performance was achieved for melanocytic lesions, but margins were difficult to detect in some cases of basal cell carcinoma. This study showed that tumor segmentation of skin PSL using HSI information is feasible, but further investigation is needed until it is implemented in clinical practice to facilitate fast and noninvasive delineation of tumor margins.

## Data Availability

Due to privacy regulations for medical information, data underlying the results presented in this paper are not publicly available at this time. However, the dataset may be obtained from the authors upon reasonable request.
